# Commitment of Scaffold Proteins in the Onco-Biology of Human Colorectal Cancer and Liver Metastases after Oxaliplatin-Based Chemotherapy

**DOI:** 10.3390/ijms18040891

**Published:** 2017-04-22

**Authors:** Deborah Rotoli, Manuel Morales, Julio Ávila, María del Carmen Maeso, María del Pino García, Ali Mobasheri, Pablo Martín-Vasallo

**Affiliations:** 1 Laboratorio de Biología del Desarrollo, UD de Bioquímica y Biología Molecular and Centro de Investigaciones Biomédicas de Canarias (CIBICAN), Universidad de La Laguna, Av. Astrofísico Sánchez s/n., 38206 La Laguna, Spain; deborah_rotoli@yahoo.it (D.R.); javila@ull.es (J.Á.); 2 CNR—National Research Council, Institute of Endocrinology and Experimental Oncology (IEOS), Via Sergio Pansini 5, 80131 Naples, Italy; 3 Service of Medical Oncology, University Hospital Nuestra Señora de Candelaria, 38010 Santa Cruz de Tenerife, Spain; mmoraleg@ull.es; 4 Service of Medical Oncology, Hospiten® Hospitals, 38001 Santa Cruz de Tenerife, Spain; 5 Service of Pathology, University Hospital Nuestra Señora de Candelaria, 38010 Santa Cruz de Tenerife, Spain; mmaefor@gmail.com; 6 Department of Pathology, Hospiten® Hospitals, 38001 Santa Cruz de Tenerife, Spain; mariadelpino.garcia@hospiten.com; 7 School of Veterinary Medicine, Faculty of Health and Medical Sciences, University of Surrey, GU2 7XH Guildford, UK; a.mobasheri@surrey.ac.uk; 8 Center of Excellence in Genomic Medicine Research (CEGMR), King Fahd Medical Research Center (KFMRC), Faculty of Applied Medical Sciences, King AbdulAziz University, 21589 Jeddah, Saudi Arabia

**Keywords:** colorectal cancer, scaffold proteins, AmotL2, FKBP51, IQGAP1, metastasized liver, FOLFOX, oxaliplatin, pericytes, telocytes

## Abstract

Scaffold proteins play pivotal roles in the regulation of signaling pathways, integrating external and internal stimuli to various cellular outputs. We report the pattern of cellular and subcellular expression of scaffoldins angiomotin-like 2 (AmotL2), FK506 binding protein 5 (FKBP51) and IQ motif containing GTPase-activating protein 1 (IQGAP1) in colorectal cancer (CRC) and metastases in liver resected after oxaliplatin-based chemotherapy (CT). Positive immunostaining for the three scaffoldins was found in most cells in healthy colon, tumor, healthy liver and metastasized liver. The patterns of expression of AmotL2, FKBP51 and IQGAP1 show the greatest variability in immune system cells and neurons and glia cells and the least in blood vessel cells. The simultaneous subcellular localization in tumor cells and other cell types within the tumor suggest an involvement of these three scaffoldins in cancer biology, including a role in Epithelial Mesenchymal Transition. The display in differential localization and quantitative expression of AmotL2, FKBP51, and IQGAP1 could be used as biomarkers for more accurate tumor staging and as potential targets for anti-cancer therapeutics by blocking or slowing down their interconnecting functions. Tough further research needs to be done in order to improve these assessments.

## 1. Introduction

Scaffold proteins bring together and facilitate macromolecular interactions between multiple modular partners that are committed to a specific subcellular task, usually forming a stable complex in a peculiar subcellular localization. These superstructures integrate functions such as enzymatic pathways, cell motility, protein sorting, signaling, stabilization, plasma membrane targeting of membrane proteins, recycling and cell polarity; in fact, they are involved in cell fate, tumorigenesis, migration, tumor progression and angiogenesis [1,2,3].

Colorectal cancer (CRC) is the fourth leading cause of cancer-associated mortality, and >95% of CRCs are adenocarcinomas [4,5]. FOLFOX (FOL—Folinic acid, leucovorin, F—Fluorouracil, 5-FU) or CAPOX (CA—capecitabine)-chemotherapy of CRC includes oxaliplatin (OX) [6] and is administered in the treatment of metastatic colorectal cancers in the neo-adjuvant, adjuvant and palliative setting, with an important percentage of unwanted side effects as peripheral neuropathy [7] and sinusoidal obstruction syndrome [8]. In the search for early markers of oxaliplatin-related toxicity, we studied the differential transcriptomics in peripheral white cells (PWCs) from patients receiving oxaliplatin-based chemotherapy (CT) and found 502 genes significantly up- or down-regulated as a result of CT [9]. Among those genes, some encoding scaffold proteins presented significant changes in their expression levels.

In our screening [9], after three cycles of oxaliplatin-based CT, the expression levels of the genes in PWCs varied as follows: *AmotL2* 3.5 times higher, *FKBP51* 2.76 times lower and *IQGAP1* with no detected pre-CT expression level to 229.5 relative to actin expression level [9].

AmotL2 (angiomotin-like 2) is a member of the angiomotin protein family responsible for maintaining cell-cell interactions to keep asymmetrical apical-basal polarity, avoiding endothelial detachment and promoting vascular tube formation. Human AmotL2 encodes two isoforms of a molecular mass of 100 and 60 kDa [10]. Most human cancers have an epithelial origin and the assessment of malignancy is based on the loss of apicalbasal polarity of the epithelial organization (epithelial mesenchymal transition (EMT)); however, whether this is a cause or consequence of tumor progression has yet to be established [11]. Loss of polarity, EMT and angiogenesis are crucial in CRC.

The immunophilin protein FKBP51 (FK506 binding protein 5) is a member of the peptidyl-prolyl isomerases (PPIs) superfamily [12]. This superfamily includes three distinct classes: the FK506-binding proteins (FKBPs) (e.g., FKBP12, FKBP51, and FKBP52), the CyclosporinA-binding proteins and the parvulin-like PPIs [13]. PPIs catalyze the *cis-trans* conversion of peptidylprolyl imide bonds in target proteins [14]. FKBP51 protein is localized in mitochondria, cytoplasm and nucleus [14,15,16]; is involved in the regulation of a variety of signaling pathways; and is considered as a molecular integrant of the adaptation process [17]. In many different tumors, altered expression levels have been described [18,19]. Through its influence on steroid receptor maturation, and the regulation of PKA [20], NF-κB [21], Akt [22] and transforming growth factor β (TGF β) [23] signaling pathways, FKBP51 plays an important role in tumorigenesis and response to anti-neoplastic therapy [24,25].

IQGAP1 (IQ motif containing GTPase-activating protein 1) is a multidomain protein ubiquitously expressed and the most versatile of the three here studied [26]. IQGAP1 modulates several cellular functions, i.e., cell cycle, cell morphology, motility, by linking elements of the cytoskeleton to cell adhesion and other signaling molecules [27,28], facilitating the space-time organization and the coordinated activation of structural and signaling molecules [29,30]. Because of this association with molecular partners, IQGAP1 accumulates in the plasma membrane at the invasive front in several cancer types [31,32,33,34,35].

**Figure 1:**
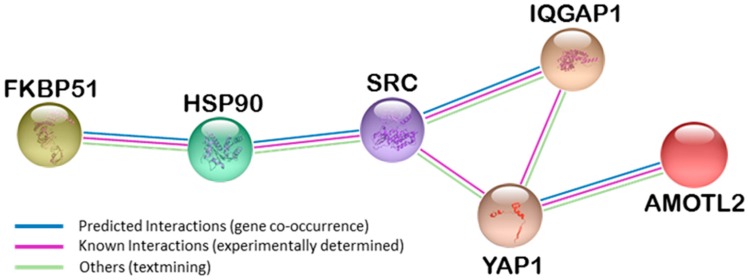
String analysis showing interactions among AmotL2, FKBP51 and IQGAP1 proteins. FKBP51 (FK506 binding protein 5); HSP90 (heat-shock protein 90); SRC (proto-oncogene tyrosine-protein kinase); IQGAP1 (IQ-motif containing GTPase-activating protein 1); YAP1 (yes associated protein); AmotL2 (angiomotin-like 2).

In Figure 1, String analysis [36] shows experimental and databases evidences of known functional interactions among AmotL2, FKBP51 and IQGAP1 in several physiological and pathological situations through Yap1, Src and HSP90. The oncogene Src and the chaperone HSP90 are well-known genes and proteins. In zebrafish embryo, AmotL2 regulates the translocation of phosphorylated Src to peripheral cell–matrix adhesion sites [37], also required for proper architecture of actin filaments. Transcriptional coactivator YAP1 (Yes-associated protein 1) (Available online: http://www.uniprot.org/uniprot/P46937) can act both as a coactivator and a corepressor and is the downstream regulatory target in the Hippo signaling pathway responsible for organ size control and tumor suppression by restricting proliferation and promoting apoptosis [38,39]. It also controls cell proliferation in response to cell contact, where the TEAD family are required for YAP-dependent gene expression [40]. The TEAD family of transcriptional enhancer factors, also known as TEA domain family, is also required for YAP-induced cell growth, oncogenic transformation, and epithelial-mesenchymal transition induction [41]. Some TEAD members are up-regulated in several types of cancer [42].

Here, we report on the cellular and sub-cellular localization and dynamics of three scaffold proteins, AmotL2, FKBP51 and IQGAP1 in colorectal primary tumors and their metastases and in apparently healthy areas of metastasized liver. The use of real human tumor samples, with its wide heterogeneity of cells, offers a more realistic and more dynamic insight into the onco-biology process and is superior to the use of primary cancer cells lines. Primary cancer cell lines studied in monolayer culture simply cannot replicate the tumor environment and the intricate complexities of the oncogenic process. Based on our findings, the published literature and information available in publicly accessible databases, we present a model of interactions between the proteins studied, a model that incorporates spatiotemporal interplay between the structural and signaling molecules involved in CRC tumorigenesis.

## 2. Results

### 2.1. AmotL2 in Healthy Colon and in CRC Tissue Samples

**Figure 2:**
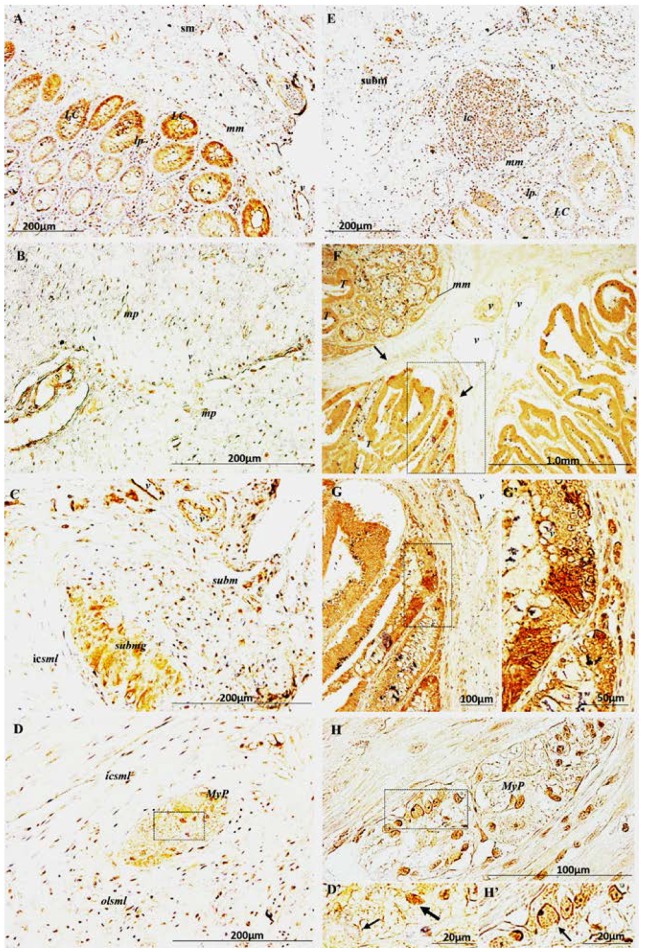
Immunolocalization of AmotL2 in: healthy colon (**A**–**D’**); and colorectal cancer (CRC) (**E**–**H’**); Healthy colon: (**A**) High intensity staining in nucleus, cytoplasm and tight junctions of epithelial cells of crypts facing the muscularis mucosae and stromal cells of lamina propria and submucosa; (**B**) Strong AmotL2^+^ labeling in nuclei of smooth muscle cells of the muscularis propria and in cells of the submucosal gland (**C**); (**D**,**D’**) AmotL2^+^ cytoplasmic expression in nerve cell bodies (thick arrow in **D’**) of myenteric plexus (**MyP**); some others are negative (thin arrow in **D’**); Immunopositive nerve fibers. CRC: (**E**) Faint immunostaining in colon epithelial cells and stronger in stromal and immune cells in the connective tissue surrounding epithelial crypts and in the submucosa; (**F**) Tumor tissue infiltrating intestinal epithelium; uniform AmotL2 staining in Lieberkühn Crypts. The intensity in the crypts facing the muscularis mucosae observed in healthy colon now is much weaker, while tumor cells exhibit a strong immunopositive staining in cytoplasm and nuclear membrane, becoming stronger at the invasive front and in budding cells (arrows); (**G**,**G’**) Magnifications of the boxed areas in (**F**,**G**), respectively; (**H**) Positive staining in neural cells of the myenteric plexus, faint in the cytoplasm and stronger in plasma membrane and nucleus (arrow in **H’**); (**D’**,**H’**) are magnifications of the boxed areas in (**D**,**H**) respectively. Strong AmotL2 immunostaining in blood vessel cells, both in healthy and tumor tissues (v). LC = Lieberkühn Crypts; lp = lamina propria; v = vessels; mm = muscularis mucosa; sbm = submucosal muscularis; sbmg = submucosal gland; ic = immune cells; mp = muscularis propria; icsml = inner circular smooth muscle layer; olsml = outer longitudinal smooth muscle layer; T = tumor lesion; MyP = myenteric plexus.

In healthy colon, AmotL2 specific staining of high intensity was observed in blood vessel cells (Figure 2A–C; v = vessels) and in stroma cells of the connective tissue surrounding Lieberkühn Crypts (LC) (Figure 2A). In the crypts, remarkable immunostaining for AmotL2 was present in epithelial cells (nucleus, cytoplasm, and tight junctions) with higher strength in the crypts facing the muscularis mucosae (mm) (Figure 2A). Positive AmotL2 labeling was also visible in nuclei of smooth muscle cells of the muscularis propria (mp) (Figure 2B) and in cells of submucosal glands (submg) (Figure 2C). Specific immunostaining for AmotL2 was also present in the cytoplasm of some nerve cell bodies of the myenteric plexus (MyP) as well as in some nerve fibers (Figure 2D,D’).

In unaffected areas of the intestinal epithelium surrounding CRC tissue samples, AmotL2 immunopositive staining was present in Lieberkühn Crypts at a lower intensity compared to healthy tissue (Figure 2E). Immune cells forming a large inflammation cluster in the submucosa showed strong labeling (Figure 2, ic = immune cells) as well as cells in the connective tissue surrounding the crypts and in endothelial cells (Figure 2, v). In tumor affected areas of the intestinal epithelium, Lieberkühn Crypts exhibited a stronger grade of AmotL2 specific staining (Figure 2F); however, the higher grade of expression in the crypts facing the muscularis mucosae observed in healthy colon was no longer visible. Immunostaining was present in tumor cells (cytoplasm and nuclear envelope) at a higher intensity in budding cells of the invasive front (Figure 2F–G’). Nerve fibers and the cytoplasm of all nerve cell bodies present in myenteric plexuses in CRC samples showed a fainter staining compared to what observed in healthy tissue, while plasma membrane and nucleus exhibited a stronger labeling (Figure 2H,H’).

### 2.2. AmotL2 in Healthy Liver and in CRC Metastasized Liver Tissue Samples

**Figure 3:**
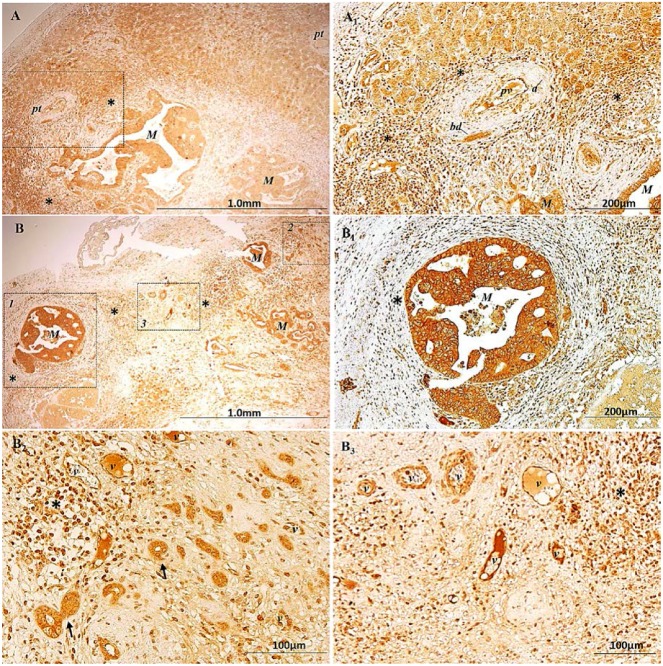
Immunolocalization of AmotL2 in CRC metastasized liver tissue samples. (**A**,**B**) Low magnification of AmotL2^+^ staining in: hepatocytes surrounding portal tracts (**A**); and malignant cells (**A**,**B**). (**A_1_**) Higher magnification of the inset in **A**; (**B_1_**) Higher magnification of the inset 1 in **B**; Asterisks identify AmotL2^+^ immune cells in the connective tissue surrounding metastasis and portal tracts. (**B_2_**,**B_3_**) Higher magnifications of the insets 2 and 3, respectively, showing the positive AmotL2 staining in nuclei of malignant budding cells (arrows in **B_2_**) and in blood vessel cells (v in **B_2_**,**B_3_**). pt = portal tract; cv = central vein; a = artery; bd = bile duct; M = metastasis; * = clusters of Amotl2^+^ immune cells.

In healthy liver, the peri-portal hepatocytes, functionally identified as zone 1 where the oxygenated blood from hepatic arteries enters, exhibited a positive AmotL2 cytoplasmic staining that increased along the way to the central vein, functionally identified as zone 3, where blood flow is less oxygenated (Figure S1A–C). This grading of staining was not further observed in apparently healthy areas of metastasized liver samples (Figure S1E,G). Moreover, in the connective tissue surrounding portal tracts in healthy liver, except for a few lymphocytes, no significant population of any inflammatory cell was observed (Figure S1D), while the connective tissue of portal tracts of CRC metastasized liver tissue appeared highly infiltrated by AmotL2^+^ immune cells, regardless of the presence or absence of malignant cells (Figure 3A,A_1_ and Figure S1H). AmotL2^+^ immune cells were also present in the connective tissue surrounding metastasis (Figure 3, asterisks). Metastasis showed high levels of AmotL2 staining in cytoplasm (Figure 3A–B_2_); in addition, in malignant budding cells the nuclei were also positive (Figure 3B_2_, arrows). In tumor associated blood vessels (v), high levels of AmotL2 specific labeling were observed in nucleus and cytoplasm of endothelial cells (Figure 3B_2_,B_3_).

### 2.3. AmotL2 in Blood Vessels

**Figure 4:**
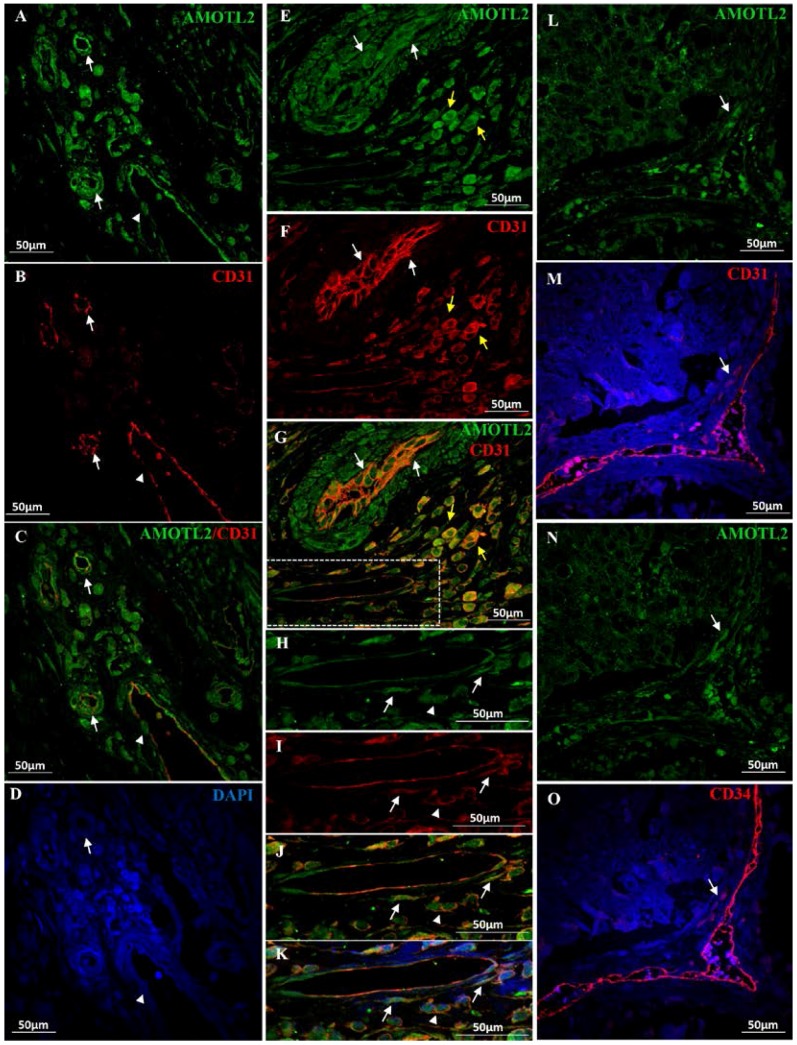
Double immunofluorescent staining of AmotL2 protein (green), the pan-macrophage and endothelial/pericyte marker CD31 (red) and the endothelial/telocyte marker CD34 in human CRC tissue sections. Cellularity is assessed with DAPI. (**A**–**D**) Blood cells of tumor associated vessels and micro-vessels (arrows) exhibit intense: AmotL2 (**A**); and CD31 (**B**) positive staining; both proteins co-localize along the vessel walls occasionally exhibiting a complementary pattern (**C**, merge). Arrowhead points to a AmotL2^+^ cell crossing the fenestrated endothelium; (**E**–**G**) Positive AmotL2/CD31staining is observed in endothelial cells (white arrows) and in macrophage-like cells surrounding the vessels (yellow arrows); (**H**–**M**) Higher magnifications of the boxed area in G showing the presence of AmotL2/CD31 positive pericytes (arrows) surrounding the blood vessel endothelium. Arrowheads point to AmotL2^+^/CD31^+^ macrophages; (**H**) AmotL2 (green); (**I**) CD31 (red); (**L**) AmotL2/CD31 merged images; and (**M**) AmotL2/CD31/DAPI merged images; (**N**–**Q**) Representative images of double labeling of: AmotL2 (green) and CD31 (red) (**N**,**O**); or AmotL2 (green) and CD34 (red) (**P**,**Q**) in serial sections from the same tissue sample, where cells co-expressing the AmotL2/CD31/CD34 proteins can be observed in perivascular areas (arrows).

In blood vessels of healthy tissues and in tumor associated blood vessels (v), high levels of AmotL2 specific labeling was observed in nucleus and cytoplasm of endothelial cells (Figure 2, v; Figure 3C,D, v; Figure 4A–G, white arrows), pericytes (Figure S1D, arrow; Figure 4H–M, white arrows) and macrophages (Figure 4E–G, yellow arrows; Figure 4H–M, white arrowhead), identified for their morphology, localization and CD31^+^ expression (Figure 4E–G, yellow arrows; Figure 4H–M, arrowhead). Figure 4N–Q shows images of double labeling of AmotL2 and CD31 (Figure 4N,O) or CD34 (Figure 4P,Q) in serial sections from the same tissue sample, where cells co-expressing the tree proteins can be observed in perivascular areas (arrows).

### 2.4. FKBP51

**Figure 5:**
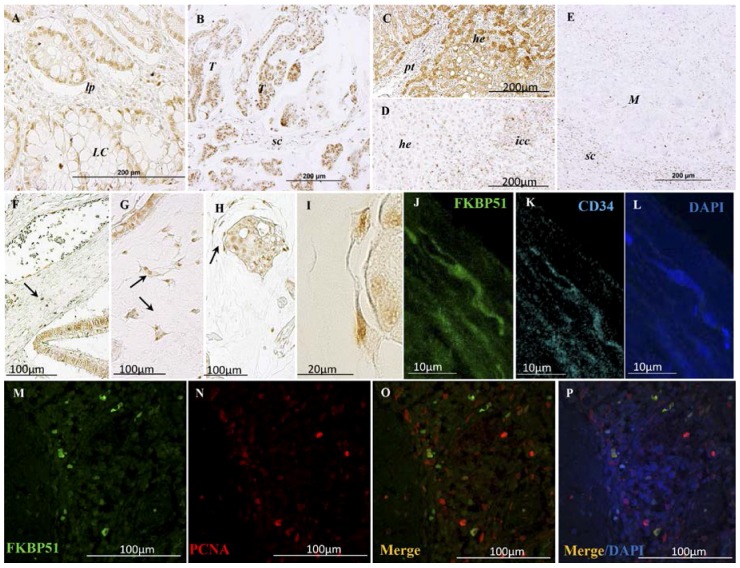
FKBP51 immunolocalization in paraffin-embedded tissue sections. (**A**) Staining is present in enterocytes (nucleus and cytoplasm) and in cells of the lamina propria in healthy colon and in unaffected areas of CRC tissue samples; (**B**) Variable positive immunostaining in nucleus and cytoplasm of CRC. Positive staining in stroma cells surrounding the lesions (sc); (**C**) Healthy liver; and (**D**) metastasized liver. Variable immunostaining in hepatocytes of both healthy liver and pathological tissue, ranging from high (**C**) to absent. In metastasized liver (**D**); in areas with high level of infiltrating immune cells (iic), cytoplasmic staining of hepatocytes is not detected, while immunostaining is localized in the nucleus; (**E**) In liver metastases, the immunostaining is faint or absent. Several stromal and inflammatory cells are FKBP51^+^. Arrow in (**F**) points to a FKBP51^+^ fibroblast showing an immature phenotype. Arrows in (**G**) point to FKBP51^+^ telocyte-like cells with typical triangular cell bodies and 2 to 5 telopodes, interconnected forming a network; (**H**) CRC tumor nest enveloped by several telocyte-like FKBP51^+^ cells (arrow); (**I**) Higher magnification of the cells pointed in (**H**); note the connection between telocyte-like cell and tumor nest; (**J**–**L**) Immunofluorescent colocalization of: FKBP51 (**J**, green); and CD34 (**K**, cyan), used as a telocyte marker. (**M**–**P**) CRC tissue section double immunostained with: FKBP51 (**M**, green); and PCNA (**N**, red); (**O**) FKBP51/CD34 merge; and (**P**) FKBP51/CD34/DAPI.

In healthy colon and in apparently healthy areas of CRC tissue sections, an intense positive FKBP51 nuclear staining in enterocytes of intestinal glands and in cells of the lamina propria was observed (Figure 5A). In CRC tissue sections, FKBP51 protein was localized in the cytoplasm and/or nucleus of tumor cells as well as in inflammatory and fibrous stromal cells surrounding the lesions (Figure 5B). Intensity of labeling varies with areas within the section, from a variable positive immunostaining in tumor cells to no staining detected in others.

In healthy liver and in unaffected areas of metastasized liver, some areas showed intense immunostaining (Figure 5C) and other well delimited a weak or absent immunostaining for FKBP51. Interestingly in high inflammatory infiltration areas of metastasized tissue sections, the cytoplasmic staining of hepatocytes was no further observed, while the protein was localized in the nucleus (Figure 5D). In metastases, the immunostaining was faint or absent, while in the inflammatory fibrous stroma several cells displayed a strong immunostaining (Figure 5E).

The presence of FKBP51 in tumor and stroma cells has been associated with the immature phenotype of stromal fibroblasts and with the EMT phenotype, suggesting a role for this protein in the EMT process [16]. Further observations allowed us to identify telocyte-like FKBP51^+^ cells located in close proximity to tumor lesions and often forming networks (Figure 5G–I), characterized by triangular or spindle body and 2–5 long, slender cytoplasmic telopodes (Figure 5G–L). Double immunofluorescent experiments using CD34 as a telocyte marker and anti-FKBP51 antibodies showed co-staining images (Figure 4J–L).

To evaluate a possible role of FKBP51 over the proliferative activity of tumor cells, the proliferation marker PCNA [43] was used in double immunofluorescence experiments on formalin-fixed, paraffin-embedded CRC samples. In several malignant cells forming tumor nests, FKBP51 and PCNA protein exhibited partial colocalization, also a few stromal cells co-expressed both. The distribution of intranuclear PCNA and FKBP51 surrounding the outer membrane of the nucleus suggests that these cells were in the early S phase (Figure 5M–P).

### 2.5. IQGAP1

**Figure 6:**
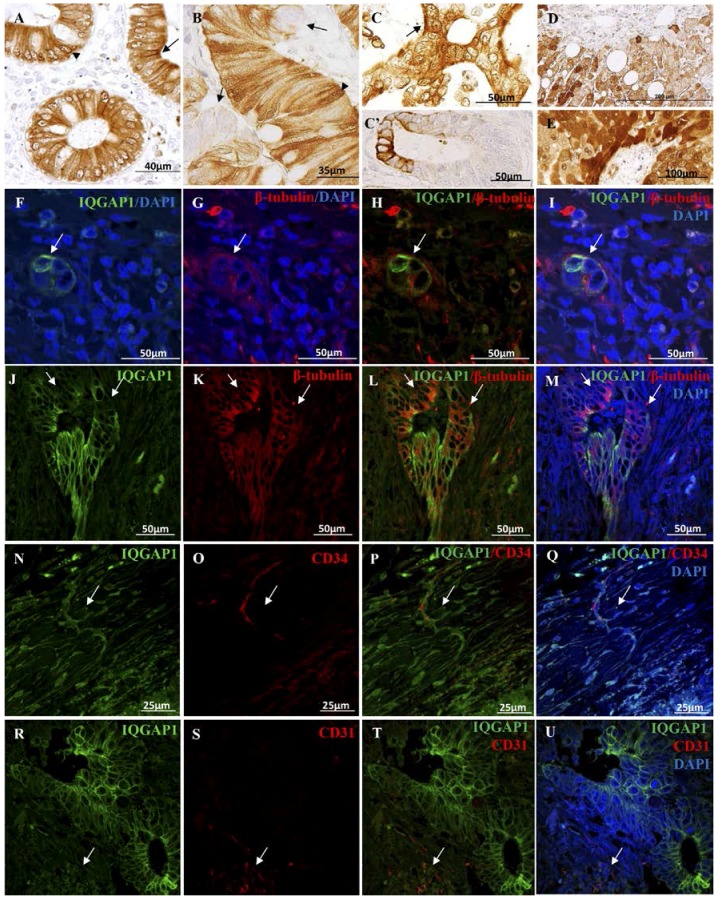
(**A**–**E**) Immunolocalization of IQGAP1 protein on paraffin-embedded tissue sections; (**A**) IQGAP1 staining in cytoplasm, apical and lateral membrane (arrow) and nuclear envelope (arrowhead) of normal epithelial cells; (**B**) Heterogeneous staining in tumor lesions, CRC IQGAP1^+^ cells (cytoplasm, lateral membrane, nuclear envelope and/or nucleus) and clusters of IQGAP1^−^ cells (arrows). Arrowhead: IQGAP1^+^ staining in nucleus; (**C**) CRC tumor lesion with strong apical staining (arrow in **C**). (**C’**) CRC tumor lesion with IQGAP1^+^ immunostaining at the invasive front.; (**D**,**E**) In healthy (**D**); and metastasized liver (**E**); heterogeneous IQGAP1^+^ staining in hepatocytes is observed (cytoplasm, nuclear envelope and/or nucleus); (**F**–**M**) IQGAP1 (green) and β-tubulin (red) double immunofluorescent staining in CRC. Variable expression/co-expression of both proteins in a budding tumor cell cluster (arrow in **F**–**I**) and in a tumor lesion (**J**–**M**). Arrows in J–M: IQGAP1^−^/β-tubulin^+^ CRC tumor cell cluster. (**N**–**Q**) Double IQGAP1 (green) and CD34 (endothelial/telocyte marker-red) on CRC. Arrow points to a CD34^+^/IQGAP1^+^ telocyte. (**R**–**U**) Co-expression of IQGAP1 (green) and CD31 (endothelial /macrophage marker-red) in several stromal cells of liver metastasis.

IQGAP1^+^ immunostaining was observed in cytoplasm, nuclear envelope, and apical and lateral membrane of normal epithelial cells (Figure 6A). In tumor lesions, CRC cells exhibited a heterogeneous staining, with clusters of IQGAP1 negative cells (arrows in Figure 5B) mixed with IQGAP1^+^ cells where the protein was localized in cytoplasm, lateral membrane, nuclear envelope and/or nucleus. High levels of labeling were also observed at the invasive front of the lesion (Figure 6C’). In many lesions, a strong apical IQGAP1^+^ immunostaining was present (Figure 6C, arrow). Hepatocytes in healthy liver and in apparently healthy areas of CRC metastasized liver (Figure 6D,E, respectively) exhibited a heterogeneous positive IQGAP1 staining in cytoplasm, nuclear envelope and/or nucleus. Assuming the role of IQGAP1 protein in the regulation of the cytoskeleton functions; double immunofluorescent experiments were performed to co-localize IQGAP1 and β-tubulin proteins in tissue specimens of CRC-affected patients (Figure 6F–M). As can be observed in Figure 6F–M, in certain areas of tumor lesions and metastasis, the co-localization of IQGAP1 and β-tubulin was lost (Figure 6J–M, arrows; Figure S2A–H).

To further characterize the localization and expression of IQGAP1 protein, we performed double immunofluorescent staining of IQGAP1 protein and the endothelial/telocyte marker CD34 or the endothelial/pericyte/macrophage marker CD31. We identified CD34^+^ telocytes (Figure 6N–Q and Figure S2I–P) and CD31^+^ stromal cells co-expressing IQGAP1 (Figure 6R–U, arrows).

Table 1 summarizes expression levels of AmotL2, FKBP51 and IQGAP1 proteins in different cells of colon and liver from healthy individuals, CRC and metastasized liver after CT. Adjuvant treatment with FOLFOX-chemotherapy was as follows: Day 1, oxaliplatin 100 mg/m^2^ intravenous (i.v.) over 2 h and leucovorin calcium 400 mg/m^2^ i.v. over 2 h; followed by 5-fluorouracil 400 mg/m^2^ i.v. bolus and by 5-fluorouracil 2400 mg/m^2^ i.v. over 46 h, every 14 days. All patients received the chemotherapy after the resection of the primary tumor. Thus, the primaries were chemotherapy naïve, while the liver metastases were chemotherapy-treated. Fifteen (27.8%) patients presented liver metastasis after CT. Curative resections of liver metastasis were performed followed by adjuvant FOLFOX-CT. The average age of patients was 59 years old (range 35–78), with 29 (54%) males and 25 (46%) females. Eight patients (15%) were stage IV and underwent palliative surgery. The other patients, T3–T4, N1–N2 (85%) were stages partial response upon RECIST (Response Evaluation Criteria In Solid Tumors) criteria. The survival is 74% (40 patients), with a follow up of six years. Localization of tumors varied from cecum (4), ascending (13), transverse (8) colon, both flexures (7), sigmoid (16) colon and sigmo-rectal (3) area and rectum (3).

## 3. Discussion

Cellularity of CRC consists of tumor cells, vascular endothelial cells and inflammatory immune cells infiltrating apparent normal colon tissue formed by mucosa, glandular, cryptal, submucosa and muscularis mucosa cells, interstitial cells, endothelial, pericytes and muscular cells of vessels and nerve cells of myenteric plexuses.

To give better information on CRC tumorigenesis and progression, instead of PCR and Western blotting methods in whole tumor pieces, for this study, we performed double immunolabeling for light and confocal microscopy in order to achieve a “cell by cell” analysis to obtain high quality subcellular localization data of AmotL2, FKBP51, and IQGAP1 proteins.

### 3.1. AmotL2 Expression in Healthy Colon and in CRC

AmotL2 localizes, virtually, in all kind of cells reported in this study but at different expression levels. Amot family includes Amot, AmotL1 and AmotL2. Amot is expressed as two different isoforms, AMOTp80 and AMOTp130 primarily localized to tight junctions [10,44]. As specified in Table 1, most cells express AmotL2 at variable levels. This fact agrees with the original report by Troyanovsky et al. [45] in several tissues and cell lines at the mRNA level, and also agrees with expression data by Microarray, RNAseq and Serial Analysis of Gene Expression (SAGE) reported by the GeneCards Human Gene Database (Available online: http://www.genecards.org/cgi-bin/carddisp.pl?gene=AMOTL2). However, little information is available on expression at the protein level. Summary in GeneCards indicates the major line of AmotL2 expression at the protein level is blood white cells. Oxaliplatin-based CT increased AmotL2-mRNA levels in white cells [9].

The over population of pericytes in vascularized areas of CRC drives to think in the possibility that some of them could come from the EMT evolvement of CRC tumor cells [46]. Most EMT cancer cells seem to be located in perivascular space and closely associated with blood vessels, thereby simulating pericytes [46,47]. It has been suggested a reprogramming of carcinoma cells into pericyte-like cells during EMT essential for tumor vascular stabilization within a new promalignant effect of EMT [46]. From this point of view, AmotL2/CD31 co-expression in pericytes and CRC cell may be indicative of a common ancestry.

CD34 is selectively expressed on hematopoietic progenitor cells and the small vessel endothelium of a variety of tissues and telocytes [48,49]. Telocytes are generally defined based on a combination of peculiar morphology, typical interstitial location, mainly within muscle fibers [48], and expression of CD34 marker (although there are no strictly telocyte-specific markers). The increased population of pericytes and telocytes as well as certain population of cells coexpressing AmotL2/CD31/CD34 suggests a pre-commitment stage of cells from which they could take different decisions of final differentiation [50].

The participation of the angiomotin family isoforms AMOTp80 and AMOTp130 in cancer cell proliferation has been studied in liver and prostate cancer [51,52]. In liver, Amot-p130 acts as a tumorigenesis facilitator when associated to Yap cofactor, while, in prostate AMOTp80, not AMOT p130, functions as a tumor promoter by enhancing PCa cell proliferation. In lung adenocarcinoma, angiomotin p130 expression correlates with poor prognosis [53].

The variation levels and the subcellular redistribution of AmotL2 shown in this study in tumor cells, metastasis and blood vessels and adjacent cells are indicative of its involvement in the CRC tumorigenesis and progression processes.

### 3.2. AmotL2 Expression in Healthy Colon, Liver and in CRC-Metastasized Liver

Most liver cells types expressed AmotL2. Hepatocytes express AmotL2, apparently, in a gradient manner, with increasing intensity as a more oxygenated zone is closer (Figure S1A,B). Our images illustrate how hypoxic gradients may contribute to CRC metastasis implantation and development in liver and how the gradients of AmotL2 expression in hepatocytes become homogeneous after being metastasized. If the gradient expression pattern disappears because of FOLFOX-CT, or if the metastasizing process also affects it, remains to be studied.

The facts that AmotL2 was originally described as a protein responsible for maintaining polarized endothelial cells attached and involved in vessel formation in angiogenesis and with a role in tumorigenesis are indicative of the complexity of association as scaffold protein, probably depending on adaptors that commit AmotL2 to biologically apparently opposite functions. Deregulated AmotL2 expression in tumor and metastasized areas during tumor progression confirms what recently has been well established in colon cancer tumors, AmotL2 expression correlates with loss of polarity by means of hypoxia activated c-Fos, leading to loss of tissue architecture [11]. The complex c-Fos/hypoxia-induced p60 and AmotL2 interacting with the Crb3 and Par3 polarity complexes retain them in large vesicles, impeding them from reaching the apical membrane [11] and being involved, this way, in EMT.

### 3.3. FKBP51 Expression in Healthy Colon, Liver, CRC and Metastasized Liver

FKBP51 protein is localized in the nuclei of enterocytes in healthy tissue while in CRC is found in nuclei and cytoplasm, exhibiting a variable range, from strong to no detectable signal. Metastases in liver show a faint or absent FKBP51 immunostaining, while the inflammatory fibrous stroma depicts several cells with a strong immunostaining. This depiction could be related to the effect of chemotherapy on tumor cells rather than with intrinsic changes of transformation of cells.

The changes of FKBP51 expression in liver tissue surrounding the metastases reported in this study, could be related with hepatic sinusoidal injury elicited by oxaliplatin therapy [54].

A role for this protein in EMT has been suggested based on the expression of FKBP51 in tumor and stromal cells. Indeed, the expression of this immunophilin has been associated to immature phenotype of the surrounding stromal fibroblasts, increased micro vessel density and tumor associated macrophages infiltration, suggesting a role in CRC process [19].

**Figure 7:**
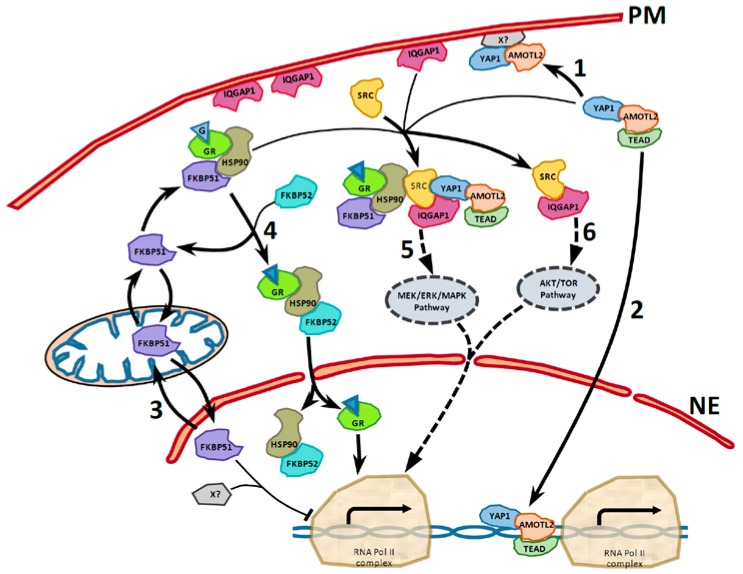
Model of direct or indirect protein-protein interaction of AmotL2, FKBP51, IQGAP2 and interacting partners. (PM) Plasma membrane. (NE) Nuclear envelope. AmotL2, as scaffold protein, may promote translocation and transcriptional activity of YAP. Upon YAP interaction with AmotL2, YAP shuttles between the cytoplasm and the nucleus. AmotL2 acts as a negative regulator of YAP by inducing its cytoplasmic retention or targeting it to cell junctions (**1**); Within the nucleus, YAP functions as a transcriptional coactivator of TEAD. The YAP-TEAD complex promotes the transcription of many genes that encode pro-proliferative and anti-apoptotic proteins. AmotL2 functions as a positive regulator of YAP by promoting its nuclear translocation (**2**), where AmotL2 may act as a cofactor for the transcription of a group of YAP-TEAD target genes (**2**); Both ways of translocation of FKBP51 between mitochondrial and nuclear pool. In the nucleus, FKBP51 regulates, presumably blocking, transcription of GR-target genes, and possibly other targets (**3**); Cytosolic FKBP51 interacts with glucocorticoid hormone receptor (GR) and HSP90. Upon binding, the GR heterocomplex exchanges FKBP51 for FKBP52. FKBP52-GR–chaperone complex is able to interact with dynein and walk by cytoskeletal tracts through cytosol and, through the nuclear pore, get into the nucleus, where dissociates and facilitates binding of the steroid-activated receptor to promoter sites (**4**); In its scaffolding function, IQGAP1 interacts with the MEK-ERK pathway hyperactivates ERK signaling and promotes tumor cell proliferation and survival (**5**). On the other hand, IQGAP1 interaction with the MEK-ERK pathway is dispensable in normal cell differentiation, growth, and survival [63]. IQGAP1 promotes cell division and proliferation through IQGAP1-TOR-Akt and suppresses differentiation and apoptosis driving to transformation [64].

FKBP51 and its co-isoform FKBP52 are HSP90 co-chaperones that modify steroid hormone receptor activity. Figure 6 and Figure 7 illustrate their interaction with the glucocorticoid receptor. The HSP90-FKBP52 complex co-immunoprecipitate with the glucocorticoid (GR) and the mineralocorticoid receptors and with the dynein–dynactin complex [16,55,56], indicating a retrograde movement of steroid receptors. FKBP51 is considered a negative regulator of receptor function. The glucocorticoids secretion by some CRC could be related to this pathway and have immunosuppressive functions cooperating, this way, to tumor progression [57].

Cytoplasmic FKBP51 is involved in the pro-apoptotic effects of rapamycin: over cell survival and chemoresistance of cancer cell. Rapamycin inhibits FKBP51 and though hinders NF-κB activation [58]. Mitochondrial-nuclear redistribution of FKBP51 is regulated by the PKA pathway; PKA and FKBP51 mainly colocalize in the nuclear lamina. In the nucleus, FKBP51 is retained by its interaction with the nuclear matrix and chromatin, regulating expression of target genes [20] (Figure 7, pathway 3).

### 3.4. IQGAP1 Expression in Healthy Colon, Liver, CRC and Metastasized Liver

The homogeneous distribution of IQGAP1 in normal cells shifts to a broad expression pattern, ranging from no expression to high level of expression in the whole CRC tumor cell. IQGAP1 seems to be involved in tumor progression, since its maximum expression is at the growing front of tumor, just at the apical part of the expanding cells. The absence of IQGAP1 and β-tubulin co-immunostaining shows groups of tumor cells undergoing EMT.

The interaction of IQGAP1 with cytoplasmic plus-end binding proteins CLIP-170 and APC, tethering microtubules to the actin network promotes nuclear envelope membrane dynamics [59,60]. Co-localization of IQGAP1, F-actin and β-tubulin proteins at the nuclear envelope has been reported by Johnson et al. in MCF-7 (breast cancer epithelial cells), HT29 (colon cancer epithelial cells) and NIH3T3 (non-tumor embryonic fibroblasts) cell lines [61]. Those observations suggest a role for IQGAP1 in cell cycle-associated nuclear envelope assembly/disassembly and in survival, cell growth, cytokinesis, and cell migration: key processes in tumorigenesis and carcinogenesis [61].

The IQGAP1^+^ nucleoli, often found throughout the microscopy field, suggests a correlation between IQGAP1 expression and RNA synthesis in tumor cells, which agrees with the previous report [62] in mouse oocyte nucleus, forming a ring around the nucleolus only in transcriptionally active oocytes.

### 3.5. Final Considerations and Future Research Directions

In this study, we confirm the fundamental role of the cell by cell immunohistochemical analysis in molecular oncology data interpretation, presenting data compatible with the involvement of AmotL2, FKBP51 and IQGAP1 proteins in the cellular EMT phenotype. We show evidence that the scaffoldins show variation in expression and localization at the protein level in tissue samples of pre-CT treated colorectal adenocarcinoma and in liver metastases from patients that underwent FOLFOX-CT. The co-localization of CD34 and AmotL2, FKBP51 and IQGAP1 in several vessels is indicative of a role for these three scaffoldings in tumor angiogenesis and/or in vascular invasion. Indeed, Yamaoka-Tojo et al. have recently shown IQGAP1 as a VEGFR2 binding protein in quiescent endothelial cells, playing an important role in the establishment of VE-cadherin-based cell–cell contacts, and suggested that IQGAP1 may function as a scaffold linking VEGFR2 to the β-catenin/VE-cadherin compound at the adherens junctions (AJ) [65].

Several pieces of evidence point to a key role for these proteins in the dynamics of tumor cells and angiogenesis process, including expression in pericytes and/or telocytes. Variations in cellular expression here described renders scaffoldins AmotL2, FKBP51 and IQGAP1 an attractive group as biomarkers for diagnostic staging and as targets for therapy, although further research needs to be done to confirm and to precise these assessments.

Figure 7 shows a model of interactions of AmotL2, FKBP51, and IQGAP1 made upon integration of our data with the literature and other data from databanks. Scaffold proteins connect structural and signaling molecules in the spatiotemporal organization and activation in CRC tumorigenic cells [29,30]. The process takes place in different subcellular localizations and at variable expression levels depending on the status of the cell within the tumor. Further studies are needed to confirm the possible existence of the complex FKBP51-HSP90-SRC-YAP-AmotL2-IQGAP.

## 4. Materials and Methods

### 4.1. Patients, Tumor Tissue and Controls

The study was approved by the Ethics Committee of La Laguna University (La Laguna, Canary Islands, Spain) and the Ethical Committee of Nuestra Señora de Candelaria University Hospital (HUNSC); Santa Cruz de Tenerife, Canary Islands, Spain (No. 198/2008, approved on 16 September 2008). All patients signed an informed consent for diagnosis and research on tissue specimens before entering in the project. Paraffin-embedded tissue samples from 54 patients, ensuring patient anonymity, and the corresponding clinical data were obtained from the reference medical areas of HUNSC.

Following the same ethics and consent rules, colon and liver samples were obtained from surgery partial exeresis pieces after trauma of three control males.

### 4.2. Antibodies

The following primary antibodies were used: rabbit anti-human polyclonal antibody (PAb) against IQGAP1 (#ABT186 Millipore Corporation, Temecula, CA, USA) 1:500 for IHC-P, 1:250 for IF; rabbit pAb against FKBP51 (#ab46002; Abcam, Cambridge, UK) 1.25:100 for IHC-P, 1:50 for IF; rabbit pAb against AMOTL2 (#LS-C178611; LifeSpan BioSciences, Seattle, WA, USA) 1:100 for IHC-P, 1:50 for IF; mouse monoclonal antibody clone PC10 against anti-proliferating cell nuclear antigen (anti-PCNA, #1486 772, Roche Diagnostics GmbH, Mannheim, Germany) 1:100; mouse monoclonal anti-human cluster of differentiation (CD)31 (ready-to-use; #IR610 Dako, Glostrup, Denmark); mouse monoclonal anti-human CD34 Class II Clone QBEnd10 (ready-to-use; #IR632, Dako, Glostrup, Denmark A/S); mouse monoclonal anti-β tubulin (#sc-101527 Santa Cruz Biotechnology, Dallas, TX, USA) 1:150. Secondary antibodies: fluorescein isothiocyanate (FITC)-conjugated goat pAb against rabbit IgG (#F9887; Sigma-Aldrich, St. Louis, MO, USA; dilution, 1:200); goat pAb against mouse IgG (DyLight^®^ 650; #ab97018; Abcam, dilution, 1:100); and biotin-conjugated goat pAb against rabbit IgG (H + L) (#31820; Thermo Fisher Scientific, Inc., Waltham, MA, USA; dilution, 1:300).

### 4.3. Immunohistochemistry

Samples were fixed in 10% formalin, for 48–72 h at 4 °C. Immunoperoxidase staining of paraffin-embedded tissue sections was performed using the avidin-biotin reaction. Briefly, 5-μm-thick tissue sections, deparaffinized in xylene and hydrated in graded alcohol baths, were autoclaved at 120 °C for 10 min in sodium citrate buffer (pH 6.0) to uncover hidden antigenic sites (antigen retrieval). Samples were then incubated for 1 h at room temperature with 5% non-fat dry milk in Tris-buffered saline (TBS) to block non-specific sites. The Avidin/Biotin Blocking kit (Vector Laboratories Inc., Burlingame, CA, USA) was used to block endogenous biotin, according to the manufacturer‘s instructions. Primary antibodies were applied to slides overnight at 4 °C. Endogenous peroxidase activity was blocked by incubating the slides with 3% hydrogen peroxidase in methanol for 15 min. Biotin-conjugated anti-rabbit secondary antibody was incubated for 2 h at 37 °C, and the specific antibody staining was amplified with the ABC Peroxidase Staining kit (Thermo Fisher Scientific, Inc.). 3,3’-diaminobenzidine substrate concentrate (#IHC-101F; Bethyl Laboratories Inc., Montgomery, TX, USA) was used to visualize immunohistochemical reactions. Samples incubated without primary antibodies were used as a negative control. Slides were counterstained with Harris hematoxylin solution DC (#253949, Panreac Química SLU, Barcelona, Spain) to visualize cell nuclei and mounted with Eukitt mounting medium (#253681, Panreac Química SLU, Barcelona, Spain). An optical light microscope (BX50; Olympus Corporation, Tokyo, Japan) was used to visualize the results of the immunostaining.

### 4.4. Double Immunofluorescence Simultaneous Staining

Immunofluorescent staining of 10% formalin-fixed paraffin-embedded tissue sections was performed as previously described [47]. Briefly, 5-μm-thick tissue sections, deparaffinized in xylene and hydrated in a graded series of alcohol baths, were autoclaved at 120 °C for 10 min in sodium citrate buffer (pH 6.0) to uncover hidden antigenic sites (antigen retrieval). Samples were then incubated for 1 h at room temperature with 5% bovine serum albumin in Tris-buffered saline (TBS) to block non-specific sites. Tissue sections were then incubated simultaneously with a mixture of two distinct primary antibodies overnight at 4 °C. Slides were then incubated for 1 h at room temperature in the dark with a mixture of two secondary antibodies raised in different species and conjugated to different fluorochromes. Slides were mounted with ProLong^®^ Diamond Anti-fade Mountant with DAPI (Molecular Probes^®^; Thermo Fisher Scientific, Inc.) to visualize cell nuclei. Slides were analyzed using Leica SP8 (Leica Microsystems, Wetzlar, Germany) confocal microscopes and Olympus FV1000 (Olympus Corporation, Tokyo, Japan).

### 4.5. Image Analysis and Statistical Analysis

To compile tables, two independent observers evaluated the specimens blindly. After an initial examination of the whole blind-coded material, cut-offs were established by consensus between each investigator. Staining intensities were graded as strong (+++), moderate (++), weak (+) or absent (−). When scorings differed by more than one unit, the observers re-evaluated the specimens to reach consensus, otherwise means of the scorings were calculated.

## 5. Conclusions

Positive immunostaining for scaffold AmotL2, FKBP51 and IQGAP1 proteins was found in most cells in healthy colon, tumor, healthy liver and metastasized liver. The expression patterns reveals the greatest variability in immune system and neural (neurons and glia) cells and the least in blood vessel cells. The simultaneous subcellular localization of these scaffoldines in tumor cells and other cell types within the tumor suggest a peculiar involvement in the onco-biology of CRC and metastasis, including a role in EMT.

## Figures and Tables

**Table 1:** Protein expression levels of AmotL2, FKBP51 and IQGAP1 in different cells of healthy colon and liver and in CRC and CRC-metastasized liver. − no expression, + faint, ++ medium, +++ high. Level/level variable level depending on area; ? indeterminate staining.

	AmotL2	IQGAP1	FKBP51
**Healthy Colon**			
Epithelial cells (*Mucosae*)	+/+++	+++	+++
Stromal cells (*Mucosae*)	+++	−	++
Immune system cells	+	?	++
Blood vessel cells	+++	++	−
Smooth muscle cells	+++	++	+
Neurous (*Myenteric plexus*)	+++	++	−/+
Glia cells (*Myenteric plexus*)	+++	++	−/+
**Colorectal Cancer**			
Tumor cells	+++	−/+++	−/++
Budding tumor cells	+++	−/+++	−/+
Tumor associated stromal cells	+++	−/++	−/+++
Epithelial cells (*Mucosae*)	++	−/+++	++
Immune system cells	+++	+++	+++
Smooth muscle cells	++	−	++
Blood vessel cells	+++	+++	−
Neurous (*Myenteric plexus*)	++	−	++
Glia cells (*Myenteric plexus*)	++	−	++
**Healthy Liver**			
Hepatocytes	+/+++	−/+++	−/++
Epithelial cells (Bile duct)	+	+	−
Blood vessel cells	+++	+++	−
Immune system cells	−	+	+
**Metastasized Liver**			
Tumor cells	+++	−/++	−/+
Budding tumor cells	+++	+++	−
Hepatocytes	++	−/+++	+/++
Epithelial cells (Bile duct)	++	++	−
Immune system cells	+++	+/++	++

## Supplementary Materials

Supplementary materials can be found at www.mdpi.com/1422-0067/18/4/891/s1.

## Abbreviations

Amot	Angiomotin
APC	Adenomatous polyposis coli
CLIP-170	Cytoplasmic linker protein CLIP-170
CT	Chemotherapy
EMT	Epithelial mesenchymal transition
FKBP	FK506 binding protein
FOLFOX	FOL—Folinic acid, leucovorin, F—Fluorouracil, 5-FU, OX—Oxaliplatin
HSP90	Heat shock protein 90
IQGAP1	IQ-motif containing GTPase activating protein 1
MAPK	Mitogen-activated protein kinase
PCNA	Proliferating cell nuclear antigen
TEAD	Transcriptional enhancer factors
YAP	Yes-associated protein
