# Supplementary material for: Commitment of Scaffold Proteins in the Onco-Biology of Human Colorectal Cancer and Liver Metastases after Oxaliplatin-Based Chemotherapy
Source: Int J Mol Sci. 2017 Apr 22;18(4):891. doi: 10.3390/ijms18040891 (PMC5412470; doi:10.3390/ijms18040891)
Supplement: Supplementary file 1 [file ijms-18-00891-s001.pdf]

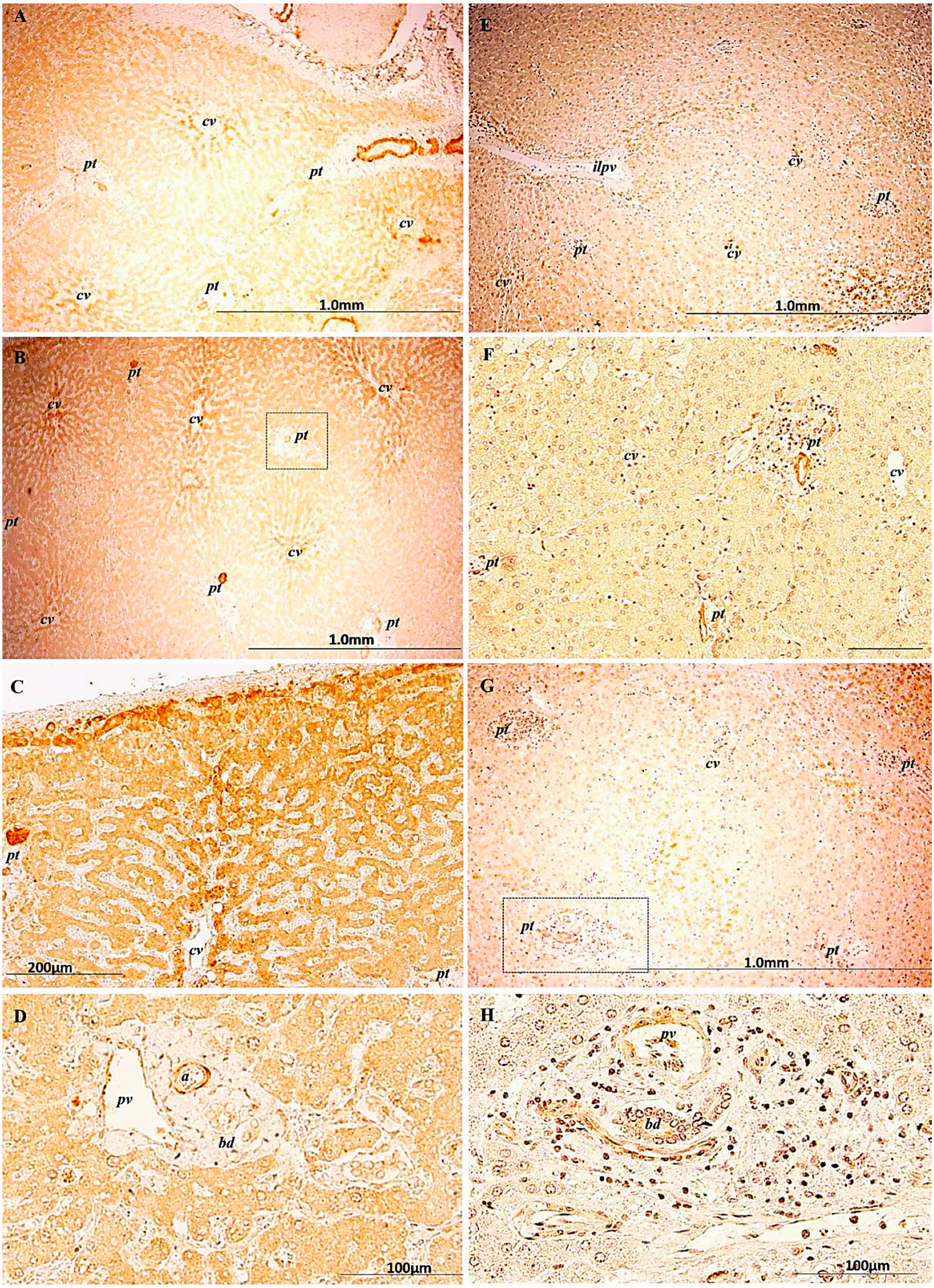

Figure S1. Immunohistochemical characterization of AmotL2 expression in healthy (A-D) and CRC metastasized liver (E-H). In healthy liver tissue sections a positive AMOTL2 cytoplasmic expression gradient is observed in hepatocytes, with

higher grades of expression in peri-venous hepatocytes (A-C). This grading of staining disappears in apparently healthy areas of metastasized liver samples (E-G). (D) and (H) are higher magnifications of the insets in B and G respectively, showing the presence of numerous inflammatory AmotL2+ cells in the connective tissue that surrounds portal tracts of metastasized CRC liver tissue samples (H), not observed in healthy liver (D). ***pt***=portal tract ***cv***=central vein ***ilpv***=inter lobular portal vein ***a***=artery ***bd***=bile duct ***M***=metastasis

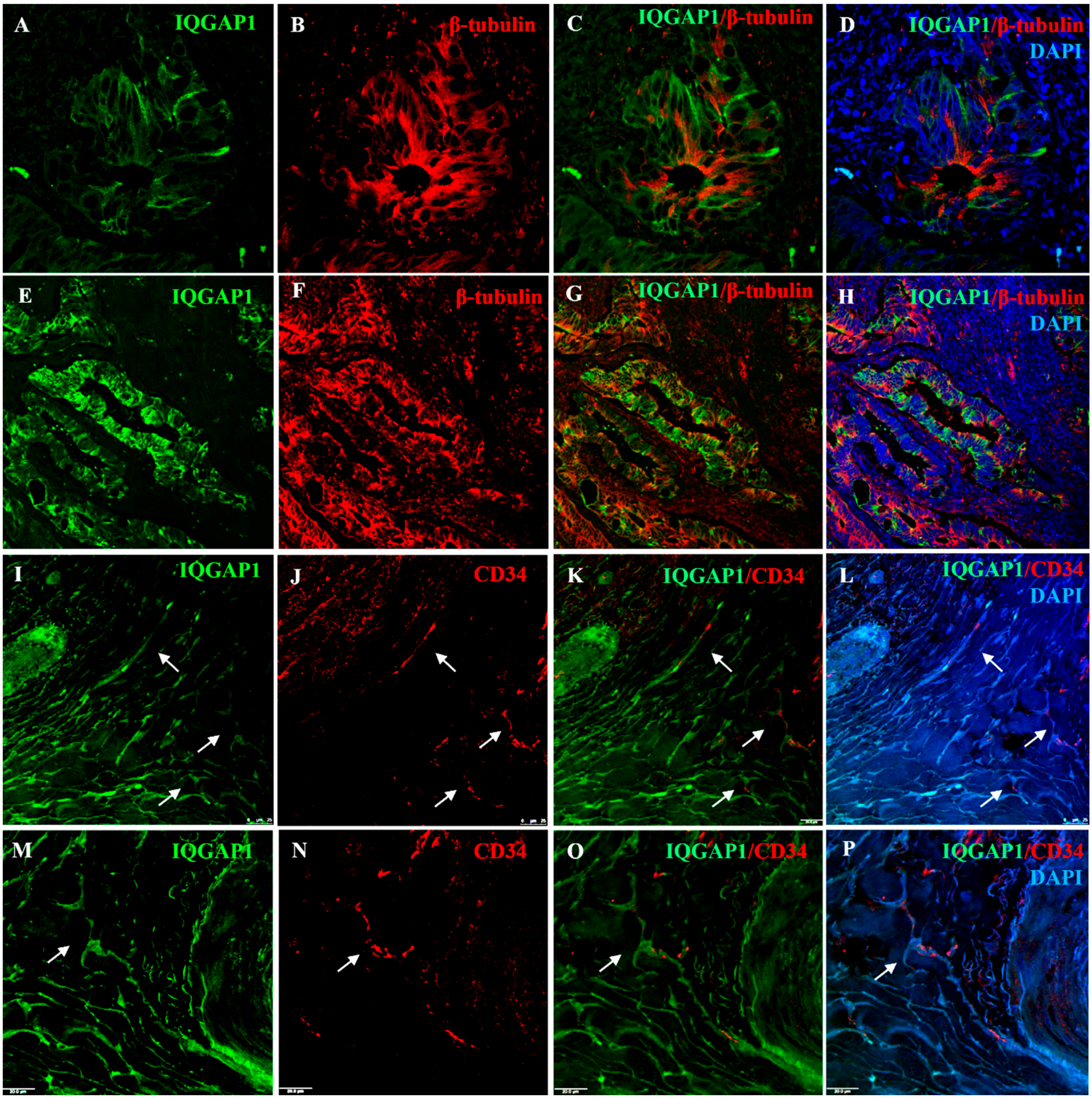

Figure S2. (**A**–**H**) Double immunolocalization of IQGAP1 (green) and β-tubulin (red) in CRC tissue samples. (**I**–**L)** Double immunolocalization of IQGAP1 (green) and CD34 (red) in CRC tissue samples. Arrows point to IQGAP1+/CD34+ telocytes. (**M**–**N**) Higher magnification of panels I–L to appreciate the peculiar morphology of telocytes (arrow).
